# DECIDER: prospective randomized multicenter phase II trial of low-dose decitabine (DAC) administered alone or in combination with the histone deacetylase inhibitor valproic acid (VPA) and all-*trans* retinoic acid (ATRA) in patients >60 years with acute myeloid leukemia who are ineligible for induction chemotherapy

**DOI:** 10.1186/s12885-015-1432-5

**Published:** 2015-05-26

**Authors:** Olga Grishina, Claudia Schmoor, Konstanze Döhner, Björn Hackanson, Beate Lubrich, Annette M. May, Caroline Cieslik, Michael J. Müller, Michael Lübbert

**Affiliations:** 1 Clinical Trials Unit, Medical Center – University of Freiburg, Elsaesser Str. 2, 79110 Freiburg, Germany; 2 Department of Internal Medicine III, University Hospital of Ulm, Albert_Einstein-Allee 23, 89081 Ulm, Germany; 3 Department of Medicine, Division Hematology/Oncology/Stem-Cell Transplantation, Medical Center – University of Freiburg, Hugstetter Str. 55, 79106 Freiburg, Germany; 4 Pharmacy, Medical Center – University of Freiburg, Hugstetter Str. 55, 79106 Freiburg, Germany; 5 Department of Pathology, Institute of Clinical Pathology, Medical Center – University of Freiburg, Ludwig-Aschoff-Haus, Breisacher Str. 115a, 79106 Freiburg, Germany

**Keywords:** Acute myeloid leukemia, Low-dose decitabine, Valproic acid, All-*trans* retinoic acid, Elderly patients

## Abstract

**Background:**

Acute myeloid leukemia (AML) is predominantly a disease of older patients with a poor long-term survival. Approval of decitabine (DAC) in the European Union (EU) in 2012 for the treatment of patients with AML ≥65 years marks the potential for hypomethylating agents in elderly AML. Nevertheless the situation is dissatisfactory and the quest for novel treatment approaches, including combination epigenetic therapy is actively ongoing. The given randomized trial should be helpful in investigating the question whether combinations of DAC with the histone deacetylase (HDAC) inhibitor valproic acid (VPA) and/or all-*trans* retinoic acid (ATRA), which *in vitro* show a very promising synergism, are superior to the DAC monotherapy. The accompanying translational research project will contribute to find surrogate molecular end points for drug efficacy and better tailor epigenetic therapy. An additional aim of the study is to investigate the prognostic value of geriatric assessments for elderly AML patients treated non-intensively.

**Methods/Design:**

DECIDER is a prospective, randomized, observer blind, parallel group, multicenter, Phase II study with a 2x2 factorial design. The primary endpoint is objective best overall response (complete remission (CR) and partial remission (PR)). The target population is AML patients aged 60 years or older and unfit for standard induction chemotherapy. Patients are randomized to one of the four treatment groups: DAC alone or in combination with VPA or ATRA or with both add-on drugs. One interim safety analysis was planned and carried out with the objective to stop early one or more of the treatment arms in case of an unacceptable death rate. This analysis showed that in all treatment arms the critical stopping rule was not reached. No important safety issues were observed. The Data Monitoring Committee (DMC) recommended continuing the study as planned. The first patient was included in December 2011. A total of 189 out of 200 planned patients were randomized since then (status 31.12.2014).

**Trial registration:**

ClinicalTrials.gov identifier: NCT00867672 (registration date 23.03.2009); German clinical trials registry number: DRKS00000733 (registration date 19.04.2011).

## Background

Acute myeloid leukemia (AML) of the older patient constitutes a major unmet clinical need since the large majority will not be found eligible for induction chemotherapy. Reasons for this decision include host factors (comorbidities, reduced performance status, functional limitations due to age), often leading to poor tolerance of repeated chemotherapy courses, as well as the unfavorable biology of this disease in older patients. Among low dose cytarabine, azacitidine or best supportive care, the hypomethylating agent (HMA) decitabine (DAC) represents one of the therapeutic options for these patients providing effectiveness, good tolerability and maintenance of quality of life. In this trial, we ask whether a combination of DAC with the histone deacetylase inhibitor VPA and/or ATRA is able to increase the objective best response rate to DAC alone in AML of the elderly.

### Rationale for combination of DAC und VPA in AML

Since the pioneering discovery by Cameron et al. [1] of a synergism between demethylation and histone deacetylase (HDAC) inhibition in the re-expression of silencing genes, investigations have been advanced in pre-clinical and clinical studies using the two DNA methyltransferase inhibitors available (azacitidine and decitabine) in combination with different HDAC inhibitors. Research of the additional effects of HDAC inhibitors appears particularly important and promising, since both mouse data [2, 3] and *ex vivo* results [4] have given clear indications that a combination of demethylation agents with HDAC inhibitors may exhibit beneficial effects on the normal hematopoiesis as well as on active suppression of the malignant clone. Three clinical studies investigated the combination of DAC and VPA in AML/MDS patients. In a large Phase II study performed at the MD Anderson Cancer Center (MDACC) [5], DAC (10 daily one hour infusions) was given concomitantly with escalating doses of VPA over 10 days to 54 patients. A response rate of 22 % was observed (10 CR, 2 CR with incomplete platelet recovery). Based on the toxicity profile, this combination was judged safe and active. Transient reversal of aberrant epigenetic marks was noted. In a study coordinated by the Ohio State University [6], 25 patients were treated with DAC for 10 days, and escalating VPA doses on days 5–21. A response rate of 44 % was reported (4 CR, 4 CR with incomplete platelet recovery, 3 PR). Overall, non-hematologic toxicity was limited, however with dose-limiting encephalopathy noted in several patients. Also in that study, epigenetic marks were studied in conjunction with treatment. Re-expression of estrogen receptor (ER) was shown to be associated with the clinical response. ER promoter demethylation, global DNA hypomethylation, depletion of DNA methyltransferase enzyme, and histone hyperacetylation were observed. A large randomized trial investigating clinical response and epigenetic modulation of DAC with or without VPA has been recently published by the MDACC [7]. The study could not show a benefit of the simultaneous addition of VPA to DAC in MDS/AML patients suggesting that different scheduling regimes have to be explored.

### Rationale for the combination of DAC and ATRA in AML

An intriguing rationale of the potential sensitization of non-M3 blasts to the effects of ATRA by pre-treatment with chemotherapy or azanucleosides has driven important lines of investigation. Several large clinical studies have addressed the role of ATRA when given in combination with high-dose chemotherapy: Schlenk and colleagues could show that the addition of ATRA to induction therapy in AML patients >60 years resulted in a survival benefit [8]. However, large studies by the Medical Research Council and MDACC did not show an additional effect of ATRA [9–11], which may be due to the timing of the dosing and/or different patient populations [12].

We have begun to address the question of a combination of DAC + ATRA by implementing a four week treatment of ATRA in a multicenter study during the second course of DAC, in patients showing sensitivity to DAC alone. Comparison of the overall survival of patients who did or did not receive ATRA during the second course did not reveal a superior outcome for patients who received ATRA [13]. The study showed feasibility of the combination DAC + ATRA in the clinical setting and comparability of its safety profile to that of DAC given alone. Furthermore, we could recently demonstrate that AML cell lines treated *in vitro* with DAC (either alone or in combination with ATRA or the HDAC inhibitor entinostat) showed enhanced *in vitro* differentiation in the presence of the retinoid, providing an additional rationale to combine ATRA with the HMA DAC [14]. Thus, taken together these data suggest that a controlled setting is needed to investigate this research question accurately.

### Rationale for the combination of VPA and ATRA in AML

Preclinical results of several groups have demonstrated that VPA alters the sensitivity towards ATRA, providing a strong rationale to advance this approach also clinically [15, 16]. Several groups combined VPA with ATRA in the treatment of MDS and AML [16–18] and showed good tolerability of this combination. In the study of the MDACC a total of 53 patients with high-risk MDS or refractory/relapsed AML were treated with a three-drug combination: azacitidine, VPA and ATRA. The overall response rate was 42 %. In previously untreated older patients, the response rate was 52 %. The treatment was shown to be safe and associated with induction of global DNA hypomethylation and histone acetylation [19]. However, the role of the drug combinations has not been tested in a rigorous fashion using a randomized design. This appears important in the context of a study with DAC, since the available evidence suggests that combinations of this HMA with a second epigenetic drug and an inducer of differentiation may reveal efficacy that is superior to the HMA alone.

Thus, in this trial, we ask whether a combination of DAC with the HDAC VPA or ATRA or with both add-on drugs is able to increase the objective best response rate to DAC alone in AML of the elderly. The hypothesis that the combination is more active than single-agent DAC will be investigated in a randomized setting.

## Methods/Design

### Study design and setting

This is a prospective, randomized, observer blind, active control, parallel group, multicenter, phase II study. The objective of the trial is the investigation of efficacy and safety of the histone deacetylase inhibitor (VPA) and all-*trans* retinoic acid (ATRA) as add-on to the epigenetically active drug decitabine (DAC) in older and unfit acute myeloid leukemia (AML) patients.

The trial has a 2x2 design randomizing the patients to the following four treatment arms: DAC, DAC + VPA, DAC + ATRA, DAC + VPA + ATRA. The effect of VPA will be investigated by comparing the combined treatment arms (DAC + VPA) and (DAC + VPA + ATRA) as experimental group versus the combined treatment arms (DAC) and (DAC + ATRA) as control group, and the effect of ATRA will be investigated by comparing the combined treatment arms (DAC + ATRA) and (DAC + VPA + ATRA) as experimental group versus the combined treatment arms (DAC) and (DAC + VPA) as control group.

Trial sites are located in Aachen, Berlin, Bochum, Braunschweig, Bremen, Düsseldorf (two sites), Esslingen, Frankfurt, Freiburg, Hagen, Halle, Hamm, Hannover, Jena, Koblenz, Lahr, Lebach, Leipzig, Lüdenscheid, Marburg, München, Münster, Offenburg, Ravensburg, Tübingen, Ulm, and Villingen-Schwenningen.

The study and all participating sites were approved by the central ethics committee (University of Freiburg, 76/10) and the respective local ethics committees. Patient’s written consent to participate in this clinical trial and translational research program was obtained prior to any study-specific procedures. The identifying number of the DECIDER trial in ClinicalTrials.gov registry is NCT00867672 and in German clinical trials registry DRKS00000733.

### Study population

The target population of this trial represents patients older than 60 years with AML according to WHO (≥20 % blasts in the peripheral blood or bone marrow) not qualifying for, or not consenting to, standard remission-induction chemotherapy or immediate allografting. Key inclusion criteria are: patient’s written informed consent; 60 years or older at time of informed consent; patients with primary or secondary AML according to WHO who are not expected to benefit from standard remission-induction chemotherapy; <30.000 leukocytes/μl and performance status ECOG 0, 1, 2. Key exclusion criteria are: Acute promyelocytic leukemia (APL), AML of FAB subtype M3; previous remission-induction chemotherapy for MDS or AML, previous allografting; previous treatment with DAC, 5-azacytidine, VPA or another histone deacetylase inhibitor, or ATRA; “low-dose” chemotherapy (e.g., hydroxyurea, cytarabine (Ara-C), melphalan, clofarabine etc.) within four weeks prior to DAC treatment, except for cytoreduction of leukocytosis ≥30.000/μl with hydroxyurea or Ara-C as proscribed by the study protocol; treatment with tyrosine kinase inhibitors, immunomodulating agents or other investigational AML treatment within the last four weeks or in a time period of drug half-life x 5 (whatever is shorter) before the first administration of DAC; treatment with cytokines within the previous four weeks. Further exclusion criteria are related to general medical conditions or contraindications to administration of investigational products according to Summary of Product Characteristics.

### Study treatment and procedures

**Fig. 1:**
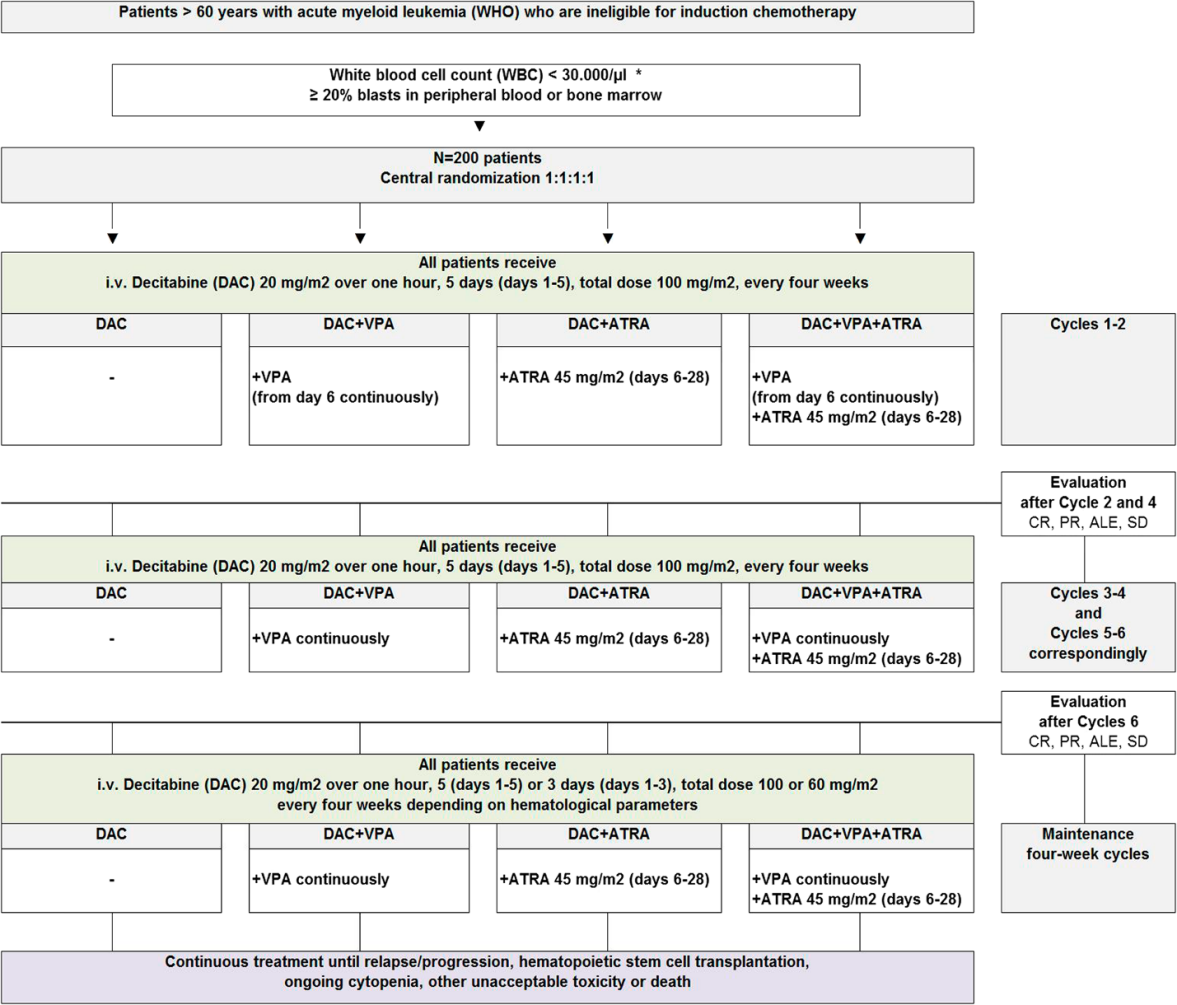
Trial flow. *If WBC ≥ 30.000/μl: Hydroxyurea (HU) or Ara-C until WBC < 30.000/μl. CR = complete remission; PR = partial remission; ALE = antileukemic effect; SD = stable disease

Study treatment in the four study arms Fig. 1 is as follows: (DAC) intravenous decitabine 20 mg/m^2^ over 1 h, 5 days (total dose 100 mg/m^2^), repeated every 4 weeks; (DAC + VPA) intravenous decitabine 20 mg/m^2^ over 1 h, 5 days (total dose 100 mg/m^2^), repeated every 4 weeks, and VPA (p.o.) from day 6 of first cycle continuously throughout all treatment cycles in a dose to achieve a VPA serum level between 50 to 110 mg/l; (DAC + ATRA) intravenous decitabine 20 mg/m^2^ over 1 h, 5 days (total dose 100 mg/m^2^), repeated every 4 weeks and ATRA (45 mg/m^2^ p.o.) from day 6 to day 28 of each treatment cycle; (DAC + VPA + ATRA) intravenous decitabine 20 mg/m^2^ over 1 h, 5 days (total dose 100 mg/m^2^), repeated every 4 weeks and VPA (p.o.) from day 6 continuously throughout all treatment cycles in a dose to achieve a VPA serum level between 50 to 110 mg/l and ATRA (45 mg/m^2^ p.o.) from day 6 to day 28 of each treatment cycle. Treatment cycles are repeated until relapse/progression, hematopoietic stem-cell transplantation, ongoing cytopenia, other unacceptable toxicity or patient’s death. Response assessment is performed at the end of the cycles 2, 4 and 6, and thereafter every third cycle. Study visits are carried out at least once a month during the treatment and every three months after treatment discontinuation until 12 months after randomization of the last patient.

### Study endpoints

The primary endpoint is the objective best overall response, defined as complete remission (CR) or partial remission (PR). Patients are counted as objective responders if they achieve a CR or a PR. All other patients are counted as non-responders. Secondary endpoints for the evaluation of efficacy are the following: (1) the overall best response defined as CR, PR, or antileukemic effect (ALE). Patients are counted as responders if they achieve a CR, PR or ALE. All other patients are counted as non-responders. The determination of CR, PR, and ALE will be performed according to the standard criteria [20, 21], (2) the progression free survival (PFS) time from randomization until relapse/progression or death. For patients being progression-free and alive at the end of the study, the PFS time will be censored at the time of the last evaluation of bone marrow, (3) overall survival (OS) time from randomization until death of the patients. For patients being alive at the end of the study, the OS time will be censored at the time of the last visit or follow-up contact, (4) quality of life of the patients using the EORTC QLQ-C30 Quality of Life Questionnaire, (5) number of nights in hospital. Further secondary endpoint for evaluation of the treatments is safety and toxicity, which are evaluated by means of adverse events (AEs) occurring from the first administration and until 4 weeks after the last administration of the study treatment, death and laboratory assessments.

### Sample size

Sample size calculation is based on the rate of patients experiencing the primary endpoint objective best overall response (CR or PR). It is the aim of this phase II study to come to a decision whether the experimental treatments VPA and ATRA are promising treatment options being worthy of further investigation in phase III. Therefore, it is not necessary and desired to come to a definitive and rigorous statistical proof of the efficacy of VPA and ATRA in this study. Nevertheless, statistical tests on the effects of VPA, i.e., (DAC + VPA) and (DAC + VPA + ATRA) vs. (DAC) and (DAC + ATRA), and ATRA, i.e., (DAC + ATRA) and (DAC + VPA + ATRA) vs. (DAC) and (DAC + VPA), will be conducted. Both tests will be performed at a one-sided significance level of 10 %. Based on the results of our single-arm Phase II study of DAC in AML (Study 00331, NCT00866073) [22], which has recruited 235 patients in Germany, an objective best overall response rate (ORR) of about 25 % in treatment arm (DAC) is assumed. The study should have a power of 80 % to detect an increase in the ORR, when the ORR after application of VPA or ATRA is about 40 %. This corresponds to an odds ratio of 2 between the treatment arms no VPA vs. VPA and no ATRA vs. ATRA, respectively. It is further assumed that VPA and ATRA do not show an antagonistic effect. With these assumptions, 176 patients have to be included in this study. This was calculated with formula (3) in Campbell et al., 1995 [23] for the Chi square-test of the hypothesis that the difference between the rates is equal to zero, and is adequate also for analysis with logistic regression [24]. To account for the possibility of some protocol violations, 200 patients will be randomized in total (ratio of 1:1:1:1 to the four treatment arms).

### Randomization

Center-stratified block randomization with randomly varying block size is performed based on computer-generated lists. Block sizes are documented separately from the study protocol in a document not accessible to investigators. Randomization is performed centrally in order to guarantee concealment of treatment allocation. Blinding of the VPA and ATRA intake is impossible since serum levels of VPA will be measured, and therefore placebo control will not be feasible. However, concealment of randomization will be guaranteed to minimize selection bias, and reviewers of response will be blinded with regard to the patient’s treatment arm to minimize assessment bias.

### Statistical analysis

The primary efficacy analysis of this clinical trial will be conducted according to the intention-to-treat principle. This means that the patients will be analyzed in the treatment arms to which they were randomized, irrespective of whether they refused or discontinued the treatment or whether other protocol violations are revealed. The analysis of the effects of the different treatment schedules (VPA vs. no VPA and ATRA vs. no ATRA) with respect to the primary endpoint objective best overall response will be performed with a logistic regression model. The treatment effects will be tested using the Wald test at a one-sided significance level of 10 %, and as estimates of the effect sizes the odds ratios will be given with 95 %-confidence intervals. A multiplicative interaction effect between VPA and ATRA will be included in the logistic regression model and its size will be estimated with 95 %-confidence interval. The analysis of the treatment effects with respect to the secondary endpoints PFS and OS will be performed with the Cox model. The analysis of the treatment effects with respect to the secondary endpoint overall best response (CR, PR and ALE) will be performed with a logistic regression model. The PFS rates and the OS rates of the different treatment arms will be estimated by the Kaplan-Meier method, and the ORR of the different treatment arms will be estimated as relative frequencies. For evaluating QOL, scores will be calculated according to Fayers et al., 2001 [25]. At the different time points during follow-up changes in relation to baseline will be calculated. With respect to the secondary endpoints QOL and number of nights in hospital, the treatment groups will be compared descriptively.

Safety is analyzed in the safety population including all randomized patients who received at least one dose of study medication, and patients are analyzed according to the received treatment. AEs are coded using Medical Dictionary for Regulatory Activities (MedDRA). The incidence of AEs is calculated as the number of patients who experienced at least one AE of a certain category as a percentage of the total number of patients in the safety population.

### Interim analysis

One interim analysis with respect to safety had to be performed three months after 22 patients have been randomized in each treatment arm. The objective of this analysis is to enable early stopping of one or more of the treatment arms in case of an unacceptable death rate. A stopping rule was established based on the following considerations: an analysis of the one arm Phase II study of DAC in AML (Study 00331, NCT00866073) showed a three-months death rate of about 35 % which is commonly regarded as acceptable. A three-months death rate of 70 % or higher is regarded as unacceptable and should lead to an early stop of the treatment arm. The error probability of early stopping if the three-months death rate is acceptable in reality (<35 %) was set as ≤5 %, and the error probability of continuing a treatment arm if the three-months death rate is unacceptable in reality (>70 %) was set as ≤5 %.

### Central hematopathology

A systematic central hematopathology review of serial bone marrow aspirates, bone marrow biopsies and matching peripheral blood smears of patients on the study is being conducted at the Institute of Clinical Pathology, University of Freiburg. The review is performed by a very experienced hematopathologist who is blinded to the treatment arm to which each patient is randomized. Written reports (patients are pseudonymized) are then sent to the local principle investigators at the respective study centers.

The review procedure includes a differential count of peripheral blood (200 cells) and bone marrow smears (500 cells) as well as histological blast quantification, including enumeration after immunohistochemistry for CD34 and CD117 on bone marrow biopsies. It serves to support the study physicians’ treatment decisions, particularly in the setting of patient relapse/progressive disease. Beyond the quality control for central response evaluation, and *post hoc* support of the cytology results of the local centers, the central hematopathology review process allows for additional, exploratory studies such as those of bone marrow fibrosis, quantification of dysplasia, enumeration of megakaryocytes, and quantification of bone marrow cellularity. These measurements, conducted in a highly standardized fashion, would not be feasible by decentralized diagnostic procedures in a multicenter trial.

The compliance of the trial sites with the central hematopathology review in sending samples is >90 %. A repository of bone marrow and blood samples will be very valuable also for future adjunct studies involving immunohistochemistry, FISH and molecular techniques.

### Central cytogenetics and molecular diagnostics

Cytogenetic and molecular genetic studies are being performed in the central reference laboratory of the German–Austrian Acute Myeloid Leukemia Study Group (AMLSG), at the University of Ulm. For conventional cytogenetic analysis, standard techniques are used and chromosomal abnormalities are described according to the International System for Cytogenetic Nomenclature [26]. The adherence in sending samples to the central laboratory is >95 % (in DECIDER sites which are members of the AMLSG) and informative karyotypes are obtained in >90 % of the patients. In addition, diagnostic samples from all patients are screened for the recurring gene fusions PML/RARA, CBFB/MYH11, and RUNX1/RUNX1T1 as well as for the presence of mutations in the genes encoding FLT3 (i.e., the ITD and TKD mutations at codons D835 and I836) CEBPA and NPM1. In analogy, success rate of molecular genetic analysis is >95 %; study centers are being informed on molecular screening results within a time window of 48 h.

### Central pharmacy

The central pharmacy of the Medical Center – University of Freiburg has a very long-standing experience in the preparation and handling of DAC. Senior staff members of the pharmacy have been involved already in the early European phase II DAC studies since 1996. Therefore a wealth of knowledge exists in handling of the solubilized solution until infusion of this notoriously unstable nucleoside. From the very beginning of the study (before the DAC marketing authorization in the EU) the central pharmacy supported and consulted study sites on preparation of decitabine. Furthermore, the central pharmacy has the task of distributing the oral study drug VPA to the different German study sites in accordance with German laws.

### Serial assessment of patient fitness and psychological state

As in the predecessor phase II trial in elderly, non-fit AML patients receiving DAC (study 00331, NCT00866073), systematic functional assessment of the patients is being conducted prior to randomization and at several defined time points during and after the study treatment [27]. It is an additional aim of the study to investigate the prognostic value of geriatric assessments for elderly AML patients treated non-intensively. This includes determination of the activities of daily living (ADL) by application of the Barthel index, assessment of quality of life by the EORTC-C30 questionnaire, application of the Hospital and Anxiety Depression Scale (HADS), and determination of psychological resilience by application of the RS-11 questionnaire. In addition, the performance status (Eastern cooperative oncology group performance status, ECOG) of the patient is captured before and at defined time points during the treatment. Comorbidities are scored prior to treatment using the Hematopoietic Cell Transplantation-Comorbidity Index (HCT-CI). The compliance with this functional assessment is >90 %.

### Translational research program

The study provides the opportunity to address not only baseline genetic and epigenetic characteristics of the patient blasts with relation to the clinical response, but also mRNA expression and epigenetic changes induced by the treatment. Bone marrow and peripheral blood cells are procured prior to treatment within the standard diagnostic workup, and in patients consenting to serial sampling, also at defined, early and late time points during the treatment. Furthermore serum is being collected and cryopreserved for future studies on miRNA expression changes with this epigenetic treatment. Serial anticoagulated blood sample allow for cell sorting for blasts vs. T-cells as well as isolation of granulocytes (provided patients have sufficient cell numbers at the different time points).

### Quality assurance system

During the clinical trial, quality control is ensured through monitoring, auditing and supervision by the authorities, if applicable. The Clinical Trials Unit of the Medical Center - University of Freiburg, Germany, is responsible for project coordination, statistical planning and analysis, data management, clinical monitoring and pharmacovigilance. An independent DMC consisting of three hemato-oncologists and one statistician was established. It is the role of the DMC to monitor and supervise the progress of the trial (including safety data and adherence to the protocol) and to advise whether to continue, modify or stop the trial. Composition and responsibilities of the DMC, structure and content of its meetings, and its relationship to other key study team members are laid down in a separate DMC charter. The DMC members are continuously informed about study progress and safety data. The DMC gave recommendations on study conduct after the review of the extensive report about the interim analysis including data on patient recruitment, baseline characteristics, eligibility violations, treatment compliance, compliance with planned visits, completeness of follow-up, and safety separately for the different treatment arms.

### Study status

Since the start of recruitment in December 2011, a total of 189 patients were randomized in the study until 31.12.2014. The interim analysis was conducted in May 2014 by the Clinical Trials Unit, and the confidential report was made available only to the coordinating investigator, medical coordinator, and to the DMC. The interim analysis showed that in all four treatment arms the critical stopping rule concerning death within three months after randomization was not reached. Furthermore, no important safety issues have been observed that would alter the assumed benefit-risk profile. The DMC statement released in July 2014 recommended continuing the study as planned, as all DMC members unanimously agreed that at the moment there are no ethical or other concerns on further conduct of the trial.

## Discussion

Treatment options for elderly AML patients are very limited because they are less capable of tolerating intensive cytotoxic induction and post-remission chemotherapy. The add-on of VPA and/or ATRA - drugs which are widely used in hematology and have well controllable side effects - to HMA has been shown to be safe in smaller studies and to act in synergy *in vitro*. The aim of our trial is to investigate the potential benefit of addition of VPA or/and ATRA to DAC with regard to disease control and overall survival in a randomized setting. As recommended by the DMC, after examination of the patients’ data in frame of the interim safety analysis, the study will be continued as initially planned. DECIDER is the clinical trial investigating the potential benefit of adding VPA or/and ATRA to DAC in view of disease control, overall survival, safety and quality of life in elderly AML patients in a randomized setting.

## Abbreviations

AE: Adverse event
ALE: Antileukemic effect
AML: Acute myeloid leukemia
ATRA: All-*trans* retinoic acid
CR: Complete remission
DAC: Decitabine
DMC: Data monitoring committee
EORTC: European organization for research and treatment of cancer
HDAC: Histone deacetylase
HMA: Hypomethylating agent
MDS: Myelodysplastic syndrome
ORR: Objective best overall response rate
OS: Overall survival
p.o.: per os
PFS: Progression-free survival
PR: Partial remission
QOL: Quality of life
SD: Stable disease
VPA: Valproic acid
Vs.: versus
WBC: White blood cell count
WHO: World health organization

## References

[1] Cameron EE, Bachman KE, Myohanen S, Herman JG, Baylin SB (1999). Synergy of demethylation and histone deacetylase inhibition in the re-expression of genes silenced in cancer. Nat Genet.

[2] Milhem M, Mahmud N, Lavelle D, Araki H, DeSimone J, Saunthararajah Y (2004). Modification of hematopoietic stem cell fate by 5aza 2′deoxycytidine and trichostatin A. Blood.

[3] Araki H, Mahmud N, Milhem M, Nunez R, Xu M, Beam CA (2006). Expansion of human umbilical cord blood SCID-repopulating cells using chromatin-modifying agents. Exp Hematol.

[4] Bug G, Gül H, Schwarz K, Pfeifer H, Kampfmann M, Zheng X (2005). Valproic Acid Stimulates Proliferation and Self-renewal of Hematopoietic Stem Cells. Cancer Res.

[5] Garcia-Manero G, Kantarjian HM, Sanchez-Gonzalez B, Yang H, Rosner G, Verstovsek S (2006). Phase 1/2 study of the combination of 5-aza-2′-deoxycytidine with valproic acid in patients with leukemia. Blood.

[6] Blum W, Klisovic RB, Hackanson B, Liu Z, Liu S, Devine H (2007). Phase I study of decitabine alone or in combination with valproic acid in acute myeloid leukemia. J Clin Oncol.

[7] Issa J-P, Garcia-Manero G, Huang X, Cortes J, Ravandi F, Jabbour E, et al. Results of phase 2 randomized study of low-dose decitabine with or without valproic acid in patients with myelodysplastic syndrome and acute myelogenous leukemia. Cancer 2015, 121:556–561. 10.1002/cncr.29085 PMC4320000 25336333

[8] Schlenk RF, Frohling S, Hartmann F, Fischer JT, Glasmacher A, del Valle F (2004). Phase III study of all-trans retinoic acid in previously untreated patients 61 years or older with acute myeloid leukemia. Leukemia.

[9] Estey EH, Thall PF, Pierce S, Cortes J, Beran M, Kantarjian H (1999). Randomized phase II study of fludarabine + cytosine arabinoside + idarubicin +/− all-trans retinoic acid +/− granulocyte colony-stimulating factor in poor prognosis newly diagnosed acute myeloid leukemia and myelodysplastic syndrome. Blood.

[10] Burnett AK, Milligan D, Hills RK, Goldstone AH, Prentice AG, Wheatley K (2004). Does All-Transretinoic Acid (ATRA) Have a Role in Non-APL Acute Myeloid Leukaemia?: Results from 1666 Patients in Three MRC Trials. ASH Annu Meet Abstr.

[11] Milligan DW, Wheatley K, Littlewood T, Craig JIO, Burnett AK, NCRI Haematological Oncology Clinical Studies Group (2006). Fludarabine and cytosine are less effective than standard ADE chemotherapy in high-risk acute myeloid leukemia, and addition of G-CSF and ATRA are not beneficial: results of the MRC AML-HR randomized trial. Blood.

[12] Estey E (2004). Clinical trials in AML of the elderly: should we change our methodology?. Leukemia.

[13] Lübbert M, Rüter BH, Claus R, Schmoor C, Schmid M, Germing U (2012). A multicenter phase II trial of decitabine as first-line treatment for older patients with acute myeloid leukemia judged unfit for induction chemotherapy. Haematologica.

[14] Blagitko-Dorfs N, Jiang Y, Duque-Afonso J, Hiller J, Yalcin A, Greve G (2013). Epigenetic priming of AML blasts for all-trans retinoic acid-induced differentiation by the HDAC class-I selective inhibitor entinostat. PLoS One.

[15] Trus MR, Yang L, Suarez SF, Bordeleau L, Jurisica I, Minden MD (2005). The histone deacetylase inhibitor valproic acid alters sensitivity towards all trans retinoic acid in acute myeloblastic leukemia cells. Leukemia.

[16] Cimino G, Lo-Coco F, Fenu S, Travaglini L, Finolezzi E, Mancini M (2006). Sequential valproic acid/all-trans retinoic acid treatment reprograms differentiation in refractory and high-risk acute myeloid leukemia. Cancer Res.

[17] Kuendgen A, Knipp S, Fox F, Strupp C, Hildebrandt B, Steidl C (2005). Results of a phase 2 study of valproic acid alone or in combination with all-trans retinoic acid in 75 patients with myelodysplastic syndrome and relapsed or refractory acute myeloid leukemia. Ann Hematol.

[18] Bug G, Ritter M, Wassmann B, Schoch C, Heinzel T, Schwarz K (2005). Clinical trial of valproic acid and all-trans retinoic acid in patients with poor-risk acute myeloid leukemia. Cancer.

[19] Soriano AO, Yang H, Faderl S, Estrov Z, Giles F, Ravandi F (2007). Safety and clinical activity of the combination of 5-azacytidine, valproic acid, and all-trans retinoic acid in acute myeloid leukemia and myelodysplastic syndrome. Blood.

[20] Cheson BD, Bennett JM, Kopecky KJ, Buchner T, Willman CL, Estey EH (2003). Revised recommendations of the International Working Group for Diagnosis, Standardization of Response Criteria, Treatment Outcomes, and Reporting Standards for Therapeutic Trials in Acute Myeloid Leukemia. J Clin Oncol.

[21] Dohner H, Estey EH, Amadori S, Appelbaum FR, Buchner T, Burnett AK (2010). Diagnosis and management of acute myeloid leukemia in adults: recommendations from an international expert panel, on behalf of the European LeukemiaNet. Blood.

[22] Lubbert M, Ruter B, Claus R, Schmid M, Germing U, Eimermacher H (2007). Continued Low-Dose Decitabine (DAC) Is an Active First-Line Treatment in All Cytogenetic Subgroups of Older AML Patients: Results of the FR00331 Multicenter Phase II Study. ASH Annu Meet Abstr.

[23] Campbell MJ, Julious SA, Altman DG (1995). Estimating sample sizes for binary, ordered categorical, and continuous outcomes in two group comparisons. BMJ.

[24] Hsieh FY, Bloch DA, Larsen MD (1998). A simple method of sample size calculation for linear and logistic regression. Stat Med.

[25] Fayers PM, Aaronson NK, Bjordal K, Grønvold M, Curran D, Bottomley A. EORTC QLQ-C30 Scoring Manual. European Organisation for Research and Treatment of Cancer Brussels; 2001.

[26] Mitelman F (1994). An International System for Human Cytogenetic Nomenclature (1995): Recommendations of the International Standing Committee on Human Cytogenetic Nomenclature.

[27] Deschler B, Ihorst G, Platzbecker U, Germing U, März E, de Figuerido M (2013). Parameters detected by geriatric and quality of life assessment in 195 older patients with myelodysplastic syndromes and acute myeloid leukemia are highly predictive for outcome. Haematologica.

